# Analysis of the Operational Efficiency of Basic Medical Insurance for Urban and Rural Residents: Based on a Three-Stage DEA Model

**DOI:** 10.3390/ijerph192113831

**Published:** 2022-10-24

**Authors:** Tong Liu, Yufei Gao, Hui Li, Liping Zhang, Jiangjie Sun

**Affiliations:** 1 Health Management College, Anhui Medical University, Hefei 230032, China; 2 School of Marxism, Anhui Medical University, Hefei 230032, China

**Keywords:** three-stage DEA model, basic medical insurance, technical efficiency, data envelopment analysis

## Abstract

Following the integration of the urban residents’ medical insurance into the new rural cooperative medical insurance in 2016, China has now formed a basic medical insurance system with the urban workers’ basic medical insurance system and the rural residents’ basic medical insurance system as the main entities. With the development of basic medical insurance, the protection for residents is becoming more and more comprehensive, and its fund expenditure also increases, so it is necessary to research the efficiency of the medical insurance fund expenditure. This paper conducts a three-stage DEA analysis of the efficiency of basic health insurance for urban and rural residents in 31 provinces, based on a Chinese panel data from 2017 to 2020. It is found that China’s health insurance operation is still in the development stage, with four regions in the efficiency frontier and Guizhou province having the lowest efficiency value nationwide. The GDP and fiscal investment on social security effectively reduce the input redundancy in the basic health insurance operation, which contributes to the efficiency of the health insurance operation. This study further proposes suggestions and countermeasures to improve the operational efficiency of China’s basic health insurance, based on the empirical results: (1) develop the economy and broaden the financing sources; (2) improve the level of health care services and improve the efficiency driven by quality; and (3) improve the level of health insurance supervision through multiple measures.

## 1. Introduction

### 1.1. Background of the Study

The outline of the Healthy China 2030 plan points out: “Health is an inevitable requirement for the promotion of comprehensive human development, is the basic condition for economic and social development. Achievement of national health and longevity is an important symbol of national prosperity and revitalization, as well as the common aspiration of the people of all ethnic groups” [1]. The health insurance system is an important system for maintaining and promoting public health. With the development of health care, China’s basic medical insurance (BMI) continues to reform, aiming to reduce catastrophic health expenditures by increasing subsidies, reimbursements and benefits in an effort to achieve universal coverage and better health care [2,3]. In 2016, China’s State Council issued opinions on integrating urban and rural basic medical insurance systems, which integrated the two systems of the urban residents’ basic medical insurance and the new rural cooperative medical care, to establish a unified urban and rural residents’ basic medical insurance system, as a way to improve the efficiency of the medical insurance operations and to serve the people. At present, China has formed a basic medical insurance system with the urban workers’ basic medical insurance system and the rural residents’ basic medical insurance system as the main components. The basic medical insurance system has played an important role in guaranteeing the safety of the basic medical expenses of the insured patients [4].

### 1.2. Research Motivation

In recent years, China’s social security system has gradually improved and has built the world’s largest social security system. By the end of 2020, the number of insured persons in the full-caliber basic medical insurance reached 1.36 billion, and the coverage of the insured persons was stable, at more than 95%. In the same year, the total income and the total expenditure of the basic medical insurance fund were CNY 2468.6 billion and CNY 2103.2 billion, respectively [5]. The fund income and expenditure are related to the health interests of almost all insured people in China. The operation of the huge medical insurance fund is a huge issue. How efficient is the operation of the medical insurance fund? Does the economic and political environment affect the operation of medical insurance? It is of great practical importance to investigate the situation and efficiency of the use of the medical insurance funds. The topic of this paper is relatively new, and the basic medical insurance for the urban and rural residents in China has not been running for a long time. Using the DEA method to analyze and evaluate the operational efficiency, we can identify the main problems in the operational efficiency of the system, and thus provide a theoretical basis for the development of the health insurance system for government health insurance-related departments worldwide.

### 1.3. Literature Review and Contribution

An objective and scientific evaluation of the operational efficiency of basic health insurance is the essential way to improve the service. The data envelopment analysis (DEA) is an effective method to evaluate the operational efficiency, which was proposed by Chames et al. in 1978, as its basic model and it has been widely used in efficiency analyses [6,7]. Fried et al. (2002) proposed a three-stage DEA model, which incorporates the environmental and other stochastic factors into the model [8], and it has been widely used in academic studies, worldwide, to measure the various efficiencies as well. For example, Hu et al. used a three-stage DEA model to calculate the efficiency of the social security expenditures in China and concluded that the overall efficiency of the social security expenditures in China is not high, that there are regional differences, and environmental factors have a strong influence on the efficiency [9]. Yi et al. measured the spatial and temporal heterogeneity of the public health care efficiency in China, using a super-efficient three-stage SBM-DEA model and analyzed the influencing factors [10]. Li et al. used a three-stage DEA model to evaluate the operational efficiency of the basic pension insurance in 31 provinces and concluded that the operational efficiency of the basic pension insurance in China is at a high level, but there is still room for improvement. Factors, such as the GDP and the urbanization level, have a positive effect on the operational efficiency of the basic pension insurance, while the old-age dependency ratio has a significant negative effect [11].

This paper draws lessons from the literatures using the DEA method. This paper collects and processes the relevant data from the *China Health Statistics Yearbook*, the *China Statistical Yearbook* and the provincial statistical yearbooks. Using DEAP 2.1 software and FRONTIER 4.1 software (Manufactured by Department of Econometrics, University of New England Armidale, NSW, Australia), a three-stage DEA model is used to evaluate the efficiency and the empirical analysis of basic medical insurance fund operation in 31 provinces of China. The two questions that this paper attempts to answer are as follows. Question 1: What is the operational efficiency of the basic medical insurance fund for the urban and rural residents in China? Question 2: What is the impact of the environmental variables on the operational efficiency of the basic medical insurance for the urban and rural residents in China? At the same time, this paper provides suggestions for the scientific improvement of the health insurance operational efficiency through empirical research, which is conducive to improving efficiency, optimizing development paths, and saving resources.

The study also provides suggestions for improving the efficiency of the basic medical insurance operation, which is conducive to improving efficiency, optimizing the development path, saving resources, and improving people’s sense of gain and well-being.

The following is shown in this paper: the current literature visualization of the English literature for basic health insurance, the theoretical foundations related to this paper, the methodology used in this study, the model used to construct the efficiency measure, the empirical study, the discussion, and the policy recommendations.

**Figure 1:**
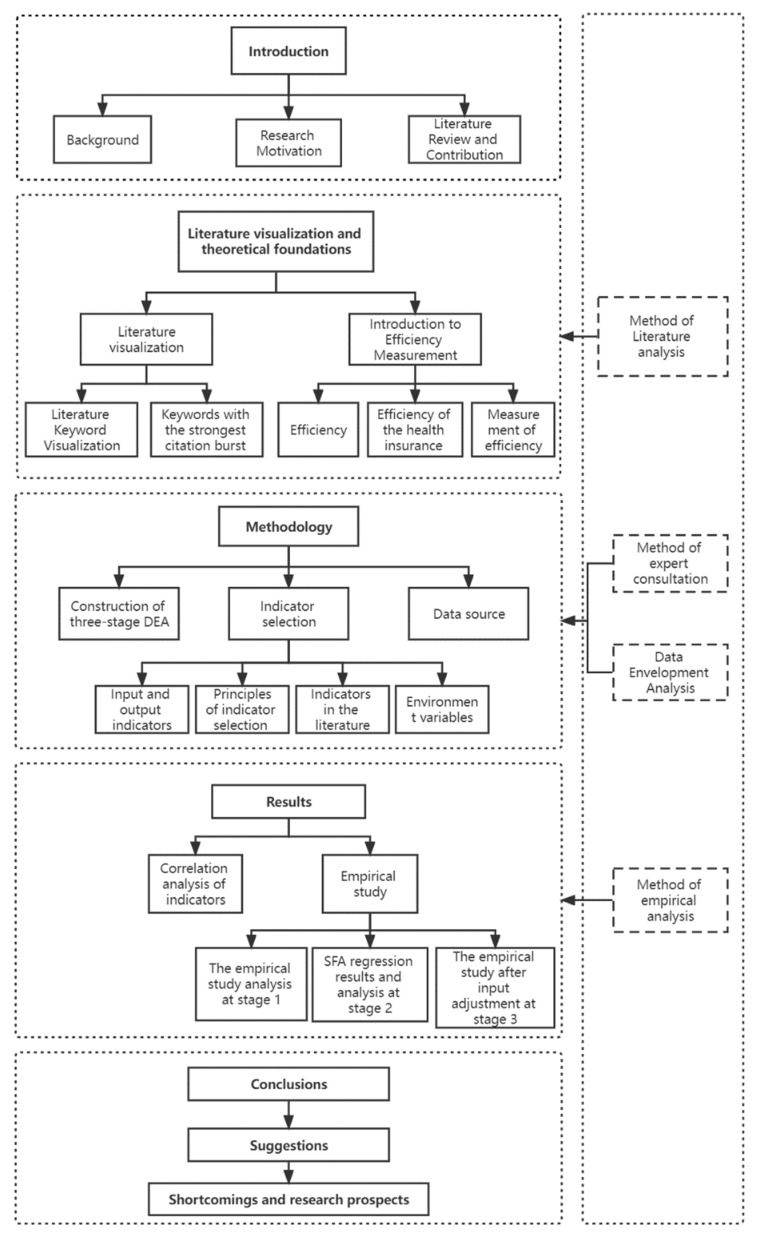
Research framework.

To facilitate the reader’s reading, the chart of the research framework is created (Figure 1).

## 2. Literature Visualization and Theoretical Foundations

### 2.1. Literature Visualization

#### 2.1.1. Visual Statistical Map of the Literature Keywords

**Figure 2:**
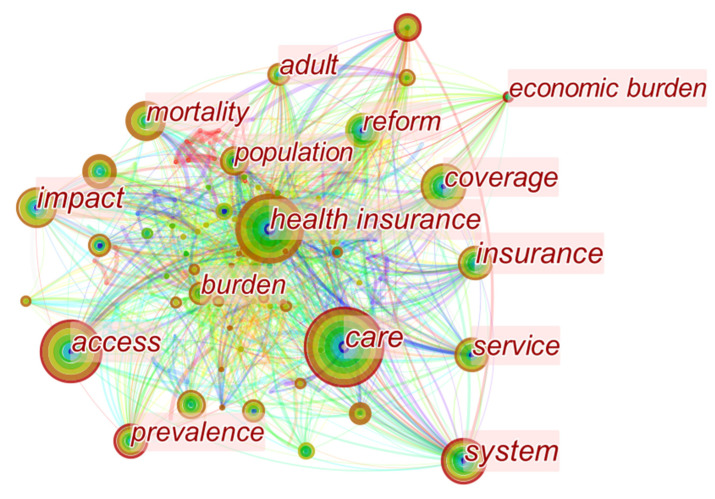
Visual mapping of the basic health insurance research on the Web of Science.

In order to understand the research situation of the basic medical insurance, internationally, we searched through 500 English articles and studies on the Web of Science core databases using “basic medical insurance” as the theme search term. We used CiteSpace software to analyze the data and to draw a visual statistical map of the literature keywords, as shown in Figure 2.

The English literature on basic medical insurance mainly concentrates on: the analysis of the current situation of health insurance and its reforms, and the empirical study of the mechanisms of action [12,13,14].

From the literature review, it can be concluded that there are few studies on the operational efficiency of basic medical insurance. This study uses the objective and reasonable efficiency evaluation tool, the three-stage DEA model, to evaluate the operation of basic medical insurance in Anhui Province, which is of great significance for analyzing the general situation of the average basic medical insurance development in China, the operational efficiency, and the influencing factors affecting the operational efficiency.

#### 2.1.2. Keywords with the Strongest Citation Burst

**Figure 3:**
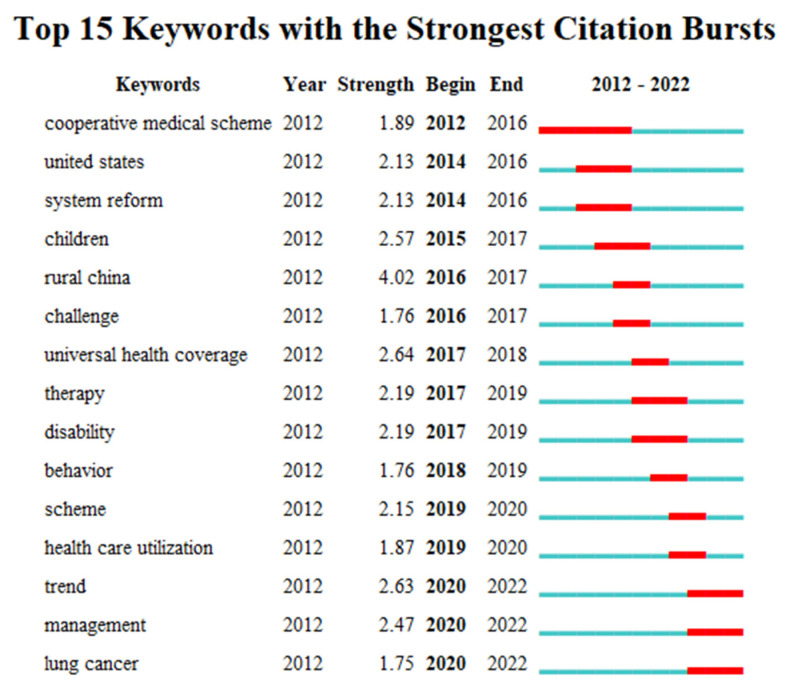
Top 15 keywords with the strongest citation burst, related to basic health insurance.

The keywords with the strongest citation burst can detect the longest lasting keywords and recent research hotspots, which are important for summarizing the research field themes and for predicting future research directions. In this paper, the top 15 keywords with the strongest citation burst in the literature and related to basic health insurance, were extracted (see Figure 3). Following the keywords analysis of the research literature from 2012 to 2022, the emergent keywords currently used include “system reform” and “health care utilization”. These are mainly related to health care management research and health care research. The keywords that are currently at the forefront of research are “trend”, “management”, and “lung cancer”, and have been for three years.

### 2.2. Introduction to the Efficiency Measurement

#### 2.2.1. Efficiency

From a cost–benefit perspective, efficiency is the ratio of inputs to outputs. The Pareto optimality, also known as the Pareto efficiency, was proposed by the Italian economist Vilfredo Pareto and refers to an ideal state of resource allocation. The process of pursuing the Pareto optimality is the process of pursuing efficient management decisions, the purpose of which is to make full use of the existing human, material, and financial resources, to optimize the allocation of the resources, and to seek to achieve the maximum efficiency and benefits.

From a management perspective, efficiency refers to the ratio relationship between the inputs and the outputs of an organization in a given time period. Efficiency in the public sector consists of two aspects: production efficiency, which refers to the average cost of producing or providing a service, and allocative efficiency, which refers to the ability of the product or service provided by the organization to satisfy the different preferences of stakeholders [15].

#### 2.2.2. Efficiency of the Health Insurance System

Medicare is not a simple mechanical repetitive process, but a complex process that requires the precise measurement of the factors of production and the prices of the factors of production, and the output of the health care services is mostly demonstrated through the patient’s condition. The Medicare Payment Advisory Commission (MedPAC) in the United States, considers the efficiency of the Medicare system as a system that produces the same or more output for less input, and as a system that can create or generate economic and social benefits [16].

In other words, the efficiency of the health care system is defined as the use of the accumulated health insurance funds through the payment of premiums by the participants, together with the financial investment of the state for health care, to achieve the optimal health care services at minimal cost, so that the social security function of the health care system is optimized and the public can feel its effectiveness at the same time [17].

#### 2.2.3. Measurement of the Efficiency

There are various modeling methods to evaluate the efficiency, including the parametric methods, such as the free distribution method, the least squared method, the stochastic frontier analysis, as well as the non-parametric methods, such as the data envelopment analysis and the price index method. The DEA (data envelopment analysis) is easier to use for the evaluation system of multiple inputs and multiple outputs, and no specific functional relationship has to be considered in the process of the arithmetic, which increases the objectivity of the study and reduces the influence of the subjective factors. The SFA (stochastic frontier analysis) is a function that includes the input, the output, and the environmental factors, which can effectively identify the inefficient and erroneous items and improve the research authenticity [8].

Fried’s three-stage DEA model integrates the DEA model with the SFA model and further incorporates the environmental and other stochastic factors into the model, which can avoid the shortcomings of the DEA that are easily disturbed by the random error terms, to make the efficiency evaluation more accurate [18].

## 3. Methodology

This research requires the analysis of efficiency through a multiple-input and multiple-output indicator system, while the complexity of the basic medical insurance operation environment requires the introduction of environmental variables to be measured and the input indicators to be reset, using measured slack variables. Therefore, the three-stage DEA model is used to study the efficiency of the basic medical insurance operation.

The initial efficiency evaluation is performed in the first stage, using the original input-output data. In the second stage, we mainly focus on the slack variables of the inputs, and regress the environmental variables and the mixed error terms with slack variables, to separate the managerial inefficiencies, the environmental effects, and the statistical noise that affect the efficiency of each decision making unit (DMU), so as to obtain a more accurate number of indicators and the environmental effects. In the third stage, the adjusted input-output variables are used to measure the efficiency of each decision unit again, and the efficiency, at this time, is reasonable and accurate through the elimination of the influence of the environmental and random factors.

### 3.1. Construction of the Three-Stage DEA

In the first stage, the input and output variables need to be entered, using the BCC model that is input-oriented and assumes the variable returns to scale. The BCC model, proposed by Banker et al. [19], calculates the technical efficiency (TE) of each DMU, which can be decomposed into the pure technical efficiency (PTE) and the scale efficiency (SE), while TE = PTE × SE. Under the assumption of the variable payoffs of the scale, the BCC model calculates more accurately, eliminates the influence of scale factors, accurately reflects the efficiency of each DMU, and yields more reasonable results.

The formula for calculating each DMU is as follows:

$$min\theta -\varepsilon \left({\hat{e}}^{T}S^{-}+e^{T}S^{+}\right)$$

(1) $$s.t.\;\left\{\begin{array}{c} \overset{n}{\underset{j=1}{\sum }}X_{j}\lambda_{j}+S^{-}=\theta X_{0}\\ \overset{n}{\underset{j=1}{\sum }}Y_{j}\lambda_{j}-S^{+}=Y_{0}\\ \lambda_{J}\geq 0,S^{-}\geq 0,S^{+}\geq 0 \end{array}\right.$$

In the equation, j = 1, 2, 3, …, n denotes the DMU, θ represents the efficiency score of each DMU, X and Y represent the input and output vectors, respectively, $S^{+}$ and $S^{-}$ represent the slack and the residual variables, respectively, $\lambda_{j}$ represents the weight of the jth DMU, and θ is a scalar, which is the technical efficiency of the DMU. The DEA model is essentially a linear programming problem.

If θ = 1, $S^{+}\;$= 0 and $S^{-}\;$= 0, the DEA of the DMU is valid.

If θ = 1, $S^{+}$ ≠ 0, $S^{-}\;$≠ 0, the DEA of the DMU is weakly valid.

If θ < 1, the DMU is non-DEA valid.

Fried [8] argues that the efficiency of the DMUs is affected by the managerial inefficiencies, the environmental effects, and the statistical noise, so it is necessary to separate these influences.

The second stage is to reduce the effects of the environmental factors and the management inefficiencies from the first stage, and to discover the factors that affect the efficiency values. Using the SFA model, the difference between the original and the desired values of the inputs, i.e., the slack variable of the inputs, was used as the explanatory variable and the environmental factors were used as the explanatory variables to establish the regression equation.

(2) $$\left\{\begin{array}{c} S_{n_{i}}=f\left(z_{i};\beta_{n}\right)+v_{n}+\mu_{ni}\\ i=1,2,3\cdots ,I\\ n=1,2,3\cdots ,N \end{array}\right.$$

In the equation, $\;S_{n_{i}}$ represents the slack variable of the nth DMU input variable i and $f\left(z_{i};\beta_{n}\right)$ represents the effect of the environmental variable $z_{i}$ with the coefficient $\beta_{n}$ on the slack variable $S_{n_{i}}$. $v_{n}+\mu_{ni}$ represents the composed error, $v_{n}$ represents the statistical noise, assuming $v_{n}$∼N(0, $\sigma_{v}^{2}$), and $\mu_{ni}$ represents the managerial inefficiency, which is assumed to follow a normal distribution function with a truncation of 0, that is $\mu_{ni}$~N^+^ (0, $\sigma_{v}^{2}$).

In this study, the method of Jondrow et al. [20] was used to decompose the composition error $v_{n}+\mu_{ni}$ with the following equation

(3) $$E\left[v_{n}|v_{n}+\mu_{ni}\right]=s_{ni}-f_{i}\left(s_{ni};\beta_{n}\right)-E\left[\mu_{ni}|v_{n}+\mu_{ni}\right]$$

The method of Dengyue L. (2012) [21] was used to calculate $E\left[\mu_{ni}|v_{n}+\mu_{ni}\right]$ in the above equation, as follows

(4) $$E\left[\mu_{ni}|v_{n}+\mu_{ni}\right]=\frac{\sigma \lambda }{1+\lambda^{2}}\left[\frac{\varphi \left(\frac{\varepsilon_{i}\lambda }{\sigma }\right)}{ø\left(\frac{\varepsilon_{i}\lambda }{\sigma }\right)}+\frac{\varepsilon_{i}\lambda }{\sigma }\right]$$

where, $\lambda =\frac{\sigma_{u}}{\sigma_{v}}$, $\varepsilon_{i}=v_{n}+\mu_{ni}$, $\sigma^{2}=\sigma_{u}^{2}+\sigma_{v}^{2}$. Φ, and ø are the density function and the distribution function of the standard normal distribution, respectively. The adjusted input values are calculated, as follows

(5) $$x_{ni}^{*}=x_{ni}+\left[max\left(z_{i}\beta_{n}\right)-z_{i}\beta_{n}\right]+\left[max\left(v_{ni}-v_{ni}\right)\right]$$

where $x_{ni}$ represents the output of each DMU before the adjustment, $x_{ni}^{*}$ represents the output of the adjusted DMU, $\left[max\left(z_{i}\beta_{n}\right)-z_{i}\beta_{n}\right]$ represents the adjustment of all DMUs to the same environmental factor, and $\left[max\left(v_{ni}-v_{ni}\right)\right]$ represents the adjustment of the random error of all DMUs to the same case.

The third stage is to replace the original input values with the adjusted input variable values calculated in the second stage and apply the DEA-BCC model to make the calculations. At this point, the values have eliminated the influence of the environmental variables, and the resulting efficiency data are more realistic and accurate.

### 3.2. Indicator Selection

#### 3.2.1. Input and Output Indicators

The reasonable selection of the input and output indicators is an important prerequisite for using the three-stage DEA model to assess the efficiency, and the establishment of the evaluation indicator system needs to follow the principles of science, refinement and comprehensiveness. This study summarizes the input-output indicators in the related literature, selects the indicators by the connotation of the C-D production function, and selects and eliminates the indicators by expert opinion to ensure the scientific and empirical accuracy of the indicators.

#### 3.2.2. Principles of the Indicator Selection

(1) Principle of comprehensiveness. Medical insurance is a complex system project, so the evaluation indicators should follow the principle of comprehensiveness. The representative and regular indicators, among the indicators reflecting the operating efficiency of the medical insurance fund, must be found using the scientific comprehensive evaluation method.
(2) Principle of objectivity. Medical insurance is a highly practical work, and its evaluation indicators system should reflect the real situation of the medical insurance fund operation. Since the economic development of each region is different and there are differences in their implemented medical insurance, the indicators with a high degree of specificity should be developed for the actual situation of each region. In order to ensure the objectivity of the evaluation indicators of the operation efficiency of the medical insurance fund, the indicators in this study are obtained from the National Bureau of Statistics, to ensure a high degree of objectivity and accuracy.
(3) Principle of operability. The evaluation indicators of the medical insurance operation efficiency should be selected to reflect the specific content of the fund operation or the indicators with easily accessible data. The accessibility and representativeness of the evaluation indicators are related to the feasibility of the evaluation indicator system. Therefore, the selected indicators should be derived from the specific work of the health insurance fund operation, taking into account the government’s and the residents’ financial and burdens, in order to ensure the sustainability of the health insurance fund operation.

#### 3.2.3. Extraction of the Indicators through the Literature Summary

This study summarizes the relevant DEA literature in health care (see Table 1). The existing literature on the health care efficiency evaluation mainly includes the input indicators: health expenditure income, number of professionals and technicians, number of insured people, etc.. The output indicators mainly include mortality, health expenditure, number of beneficiaries, etc.

In terms of the input indicators, this study follows the C-D production function, which is based on the three factors (capital factor, labor factor, technology factor) to measure the input of the health insurance operation. Thus, this study is based on the literature summary, guided by the meaning of the production function, and combined with the expert opinions, to determine the indicators in this paper. The input indicators are: fund income (capital factor), number of health technicians (labor/technology factor), and government health expenditure (capital factor), and the output indicators are: fund expenditure, epidemic morbidity rate, and resident health insurance expenditure.

#### 3.2.4. Environmental Variables

The selection of the environmental variables is an important step in the second stage of the three-stage DEA model. The selection of the indicators should be factors that meet the requirements of the “separation assumption”, can affect the efficiency of the basic health insurance operation, and will not be subjectively influenced.

Following the analysis of the operation of the basic health insurance in China, and summarizing the literature related to the efficiency evaluation, we selected three factors, namely, the per capita GDP, fiscal social security and employment expenditures, and the fiscal revenue expenditure ratio, as the environmental factors.

The GDP is the core national economic accounting index. The GDP of each region mainly reflects the gross product of each region, as well as the economic condition and development level of each city [28]. It is generally believed that the level of economic development is positively correlated with the efficiency of the health services, and a higher level of economic development can improve the operational efficiency of the basic health insurance. Conversely, the economic development may bring about the disparities between the rich and the poor, resulting in wasted resources and lowering the operational efficiency. Therefore, the GDP, which represents the level of economic development, is used as the environmental variable to analyze the operation of the health insurance in China.

The level of government finance reflects the government’s financial capacity and the support for the various undertakings. Basic medical insurance cannot operate without government financial subsidies and support, and in the same way as the economic development, the financial support has a certain influence on the operation of the medical insurance, so it is included in the environmental variables for the calculation. This study uses the social security and employment expenditure, and the fiscal revenue and expenditure ratios, to describe the level of government finance. The expenditure finance on social security and employment reflects the support of the local municipalities to the social security system, and the fiscal revenue and expenditure ratio is the degree of fiscal freedom, which reflects the ability of the local governments to use financial resources.

### 3.3. Data Source

In 2016, China established a unified basic medical insurance system for urban and rural residents, and the basic medical insurance data for the urban and rural residents were updated from 2017. Therefore, this paper selects the relevant data from the 2017–2020 *China Health Statistical Yearbook*, *China Statistical Yearbook*, and the statistical yearbooks of each region, to calculate the operational efficiency of the basic medical insurance in 31 provinces in China. The input variables, the output variables and the environmental factors are shown in Table 2.

## 4. Results

### 4.1. Correlation Analysis

Prior to the calculation of the efficiency of the basic health insurance operation, the correlation test was first performed on the input-output indicators, to ensure that there is a certain correlation between the input-output indicators. We used SPSS 22.0 software to calculate the Pearson correlation test matrix and concluded that the indicators have a high degree of correlation with each other, so the indicators, set in the earlier part of this study, can be analyzed for efficiency. The Pearson correlation matrix for the input-output data indicators is shown in Table 3.

### 4.2. Empirical Study

#### 4.2.1. The Empirical Study Analysis at Stage 1

In this study, we use the DEA-BCC model with the input-oriented and variable returns to scale in the first stage and use DEAP2.1 software to calculate the technical efficiency (TE) of 31 provinces in China, from 2017 to 2020, and the results are shown in Table 4. The values in the table are the efficiency values, and the efficiency value of 1 means that the province is in the efficiency frontier, that is, the efficiency is the best. The closer the value is to 1, the better the efficiency.

Overall, China’s basic medical insurance fund has a good operational efficiency during the sample years. The mean value of the efficiency is 0.921, but there is still improvement to be made. Among all provinces, Jiangxi, Tibet, Henan, Qinghai, and Ningxia maintain a technical efficiency of 1 over the 4-year period, meaning that they lead in terms of technical efficiency. The other provinces still have space for improvement, in terms of efficiency. Considering the economic development of these five efficiency frontier provinces, these regions are among the economically deprived regions in China, yet they have the highest health care efficiency. This may be due to the fact that the backwardness of the health care resources has instead allowed these resources to be fully utilized, and thus the health output has achieved better returns, compared to the backward health inputs.

The above table shows the preliminary calculated efficiency values of the basic medical insurance funds for the urban and rural residents across China, from 2017 to 2020. However, the data are affected by the environmental factors and the random disturbances, and there are some errors with the actual efficiency situation, and it is not possible to conclude whether the efficiency values are high or low. Therefore, it is necessary to further adjust and measure the operational efficiency of the basic medical insurance fund for urban and rural residents in China, after excluding the effects of the environmental factors and the random disturbances.

#### 4.2.2. SFA Regression Results and Analysis at Stage 2

In the second stage of the three-stage DEA model, the SFA method was used to calculate the effects of the environmental factors on the efficiency values, to reduce the errors from the first stage, and to analyze the operational efficiency of the real urban and rural residents’ basic medical insurance across China.

The deviation between the expected and actual values of the inputs calculated in the first stage, is the slack variable. Using the maximum likelihood estimation through FRONTIER 4.1 software, the slack variables of the three inputs were used as the explanatory variables, and the values of the three environmental factors, namely the GDP per capita, social security and employment expenditures of the treasury, and the fiscal revenue and expenditure ratio. The results of the SFA model operations are shown in Table 5. The significance tests found that most of the environmental variables were significant, and from the table, we can see that the different environmental variables have an effect on the relaxation variables, and it is important to exclude the effect of the environmental factors and random errors.

In analyzing the effects of the factors on the slack variables, the main analysis is the positive and negative coefficients of the environmental factors in the above table, and the positive and negative coefficients of the environmental variables are the key to influence the output. If the coefficient is positive, it means that with the growth of this environmental factor will lead to the growth of the input slack variables, i.e., the output will decrease, which is not conducive to the improvement of the efficiency of the basic medical insurance; while when the coefficient is negative, it means that the growth of this environmental factor will reduce the input slack variables, i.e., the output will increase, which is conducive to the improvement of the efficiency of the basic medical insurance operation.

**The GDP per Capita**. The calculation results show that the regression coefficients of the GDP per capita on the slack variables of each input are negative, which means that the GDP has a facilitating effect on the operation of the basic health insurance. As the economy grows, the higher economic vitality can expand the health insurance fund and increase the income of the health insurance. At the same time, the high GDP also represents the high government financial power, which can increase the health personnel and financial investment in health. Therefore, a high level of the GDP can improve the efficiency of the basic health insurance. In the context of China’s provincial economies, although there may be inefficiencies caused by wasted resources in the better developed regions, they still have a higher level of quality medical services and high-quality medical resources, which have a “siphoning” effect on the residents’ medical behavior, causing them to form medical habits that favor big cities, which can increase the medical output and improve the efficiency of the medical insurance.

**Social Security and Employment Expenditure**. The calculation results show that this factor has negative regression coefficients on each input slack variable, which means that the government social security and employment expenditures have a catalytic effect on the operation of the basic health insurance. With the increase of the government expenditure on social security and employment, the coverage rate of the health insurance can be increased, and the number of insured can be increased, which in turn can increase the income of the health insurance; at the same time, the increase of this factor will also increase the government’s investment in the health sector, so the number of health technicians will also increase accordingly, which is conducive to improving the efficiency of the health insurance operation. Some of China’s more developed provinces have high levels of social security, which facilitate the timely and effective health care for residents and improves the efficiency of the health insurance operations.

**Fiscal Revenue Expenditure Ratio**. The calculation results show that this factor has a negative regression coefficient on the slack variable of the number of health technicians and a positive regression coefficient on the slack variables of the fund income and government health expenditure. The higher the degree of fiscal freedom, the greater the government’s investment in health technicians, which is conducive to the operation of health insurance. However, the higher the degree of fiscal freedom, the higher the degree of marketization, and the market plays a dominant role in the allocation of the medical resources. As a kind of non-market redistribution and social mechanism, the efficiency of the basic medical insurance operation is affected by the high degree of the fiscal freedom of the government, which is not conducive to the level of efficiency. So it is undesirable to increase this factor blindly, and the basic medical insurance operation efficiency should be effectively improved on a moderate basis.

#### 4.2.3. The Empirical Study after the Input Adjustment at Stage 3

##### Analysis of the Operational Efficiency Value of the Basic Medical Insurance for Urban and Rural Residents in China

Following the SFA regression, the input variables were made to be in the same external environment. In the third stage of the DEA analysis, DEAP2.1 software was used to re-measure the total efficiency (TE), the pure technical efficiency (PTE), and the scale efficiency (SE) of the basic health insurance operation in 31 provinces of China, from 2017 to 2020, using the adjusted input data and the original output data. The data results are shown in Table 6 and the radar chart comparing the efficiency values before and after the adjustment, is shown in Figure 2.

**Figure 4:**
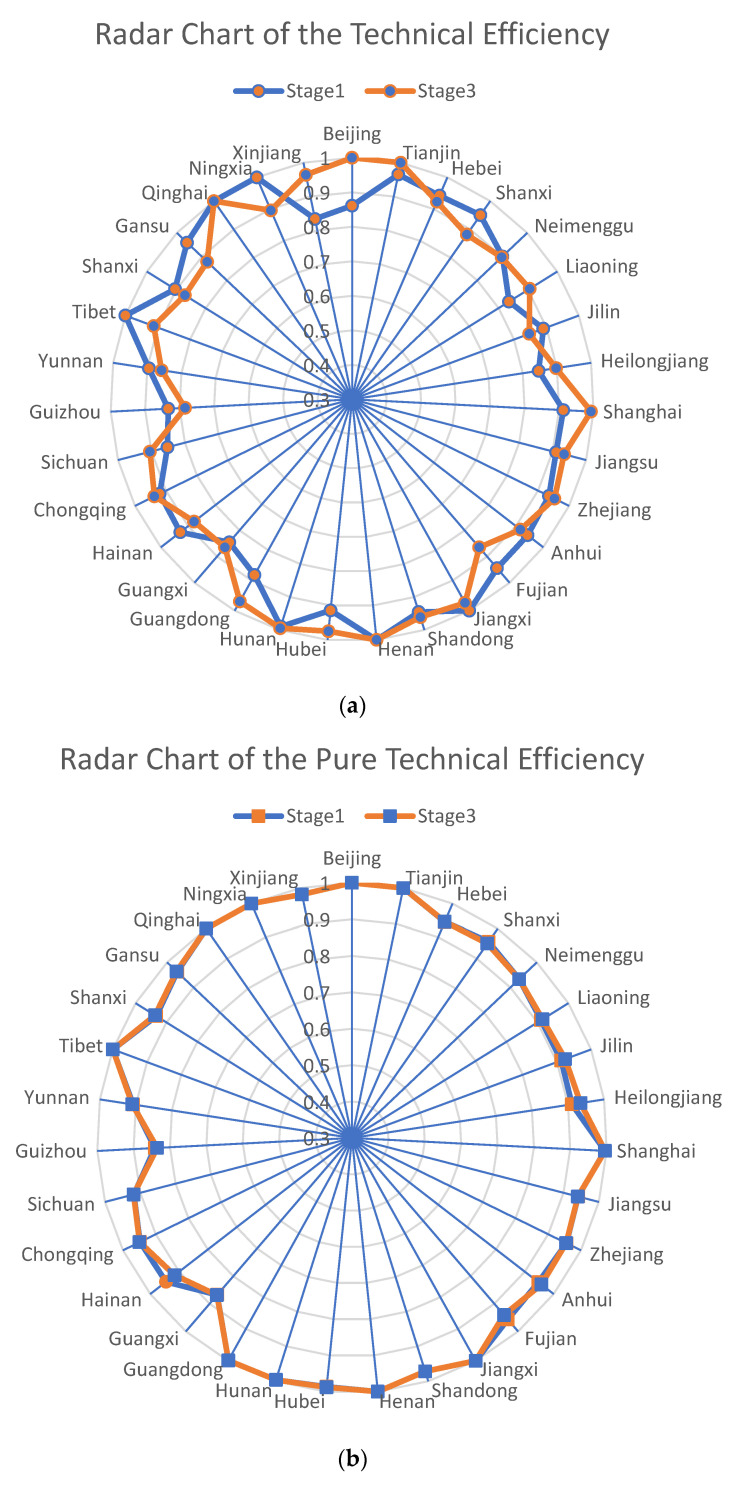

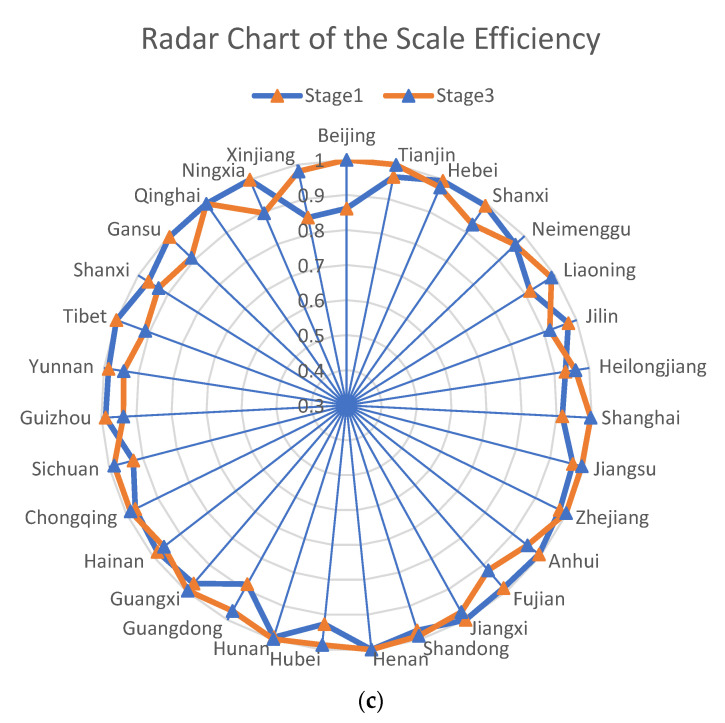
(**a**): Radar chart of the technical efficiency; (**b**): Radar chart of the pure technical efficiency; (**c**): Radar chart of the scale efficiency.

Observation the data in the table reveals that there are some differences in the measured results before and after the adjustment, after excluding the effects of the environmental factors and the random disturbances, indicating that it is meaningful to construct the SFA model for the estimation in the second stage. The differences in the data are better observed in the radar plot (Figure 4), and combined with the formula TE = PTE × SE, the efficiency of the fund operation in each region of China, is discussed next in terms of the TE, PTE, and SE, respectively.

Observation of the three radar plots reveals that the difference in the pure technical efficiency is small after excluding the effect of the environmental factors, and the difference in the technical efficiency before and after the adjustment is mainly caused by the change in the scale efficiency. This may be due to the fact that the environmental factors are associated with the size of the health insurance funds in the different regions, and adjusting for the environmental factors has a greater impact on the scale efficiency, which in turn affects the technical efficiency values.

In terms of the technical efficiency values, except for the two regions that are on the efficiency frontier, both before and after the adjustment (Henan Province and Qinghai Province), the efficiency values of all of the other regions have increased or decreased to different degrees, which also indicates that there are significant differences in the operational efficiency of the basic medical insurance funds in the different regions of China. The number of regions in the efficiency frontier (efficiency value of 1) before the adjustment, is five, and it is reduced to four, after the adjustment. This indicates that the efficiency values of Jiangxi Province, Tibet Autonomous Region, and Ningxia Autonomous Region in the first stage, are affected by the environmental factors and the management inefficiencies, which do not reflect the real situation; meanwhile, the efficiency values of Beijing and Tianjin are underestimated by the environmental factors.

##### Regional Distribution of the Technical Efficiency of the Basic Medical Insurance Funds in China

Following the comparison of the efficiency values before and after the adjustment, in order to better compare the cross-sectional differences between regions, this study analyzes the actual efficiency values of each place and draws a map of 31 provinces of China in gradient colors, according to the map of China’s provincial divisions with the adjusted efficiency values of the basic medical insurance operations, see Figure 4, where the deeper colors represent the higher efficiency values and the lighter colors represent the lower efficiency values.

**Figure 5:**
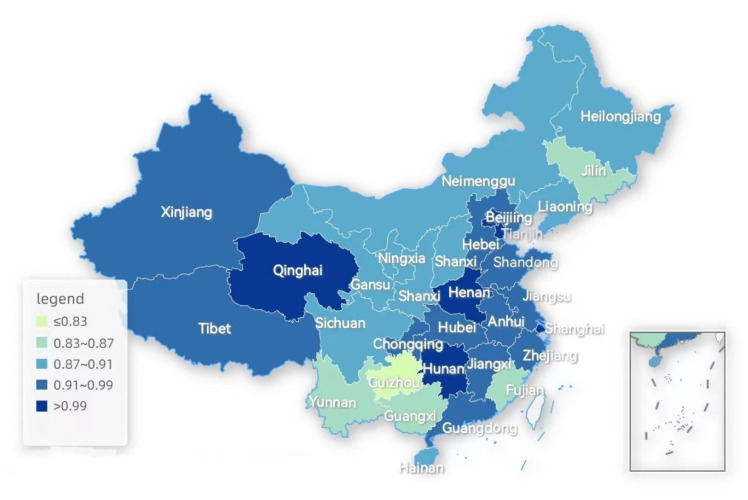
Adjusted efficiency of the basic medical insurance in 31 provinces of China.

By observing Figure 5, it is found that overall, there are fewer regions in the country at the efficiency frontier, with an uneven development of the efficiency across the country, and the high efficiency regions are concentrated in the developed eastern regions. The efficiency value of the basic medical insurance operation in Guizhou province is the lowest in China, and it needs to focus on promoting the rational allocation of resources and improving the efficiency of the medical insurance fund, in the next work arrangement. Meanwhile, Beijing, Tianjin and Shanghai, as the best developed regions in China, also have higher efficiency values, indicating that the development of the cities is conducive to the development of the health insurance business. However, Fujian, a coastal city with a better economic development, has a lower efficiency value and is not at the efficiency frontier, indicating that although the health resources are abundant there, the resources have not been allocated rationally, which also indicates that a large financial investment does not necessarily lead to an increase in the efficiency value of the basic health insurance, but may result in a waste of resources and a lower efficiency value instead. Some inland areas, such as Qinghai Province and Henan Province, are efficiency frontier areas, indicating that these two cities are effective in terms of the resource investment and can learn from such areas with a good resource allocation and efficient health insurance operation policy methods.

## 5. Conclusions

In this paper, a three-stage DEA model is used to process and analyze the data, based on which the indicators are selected through the C-D production function. Using the fund income, the number of health technicians, and the government health expenditure as the input indicators, and the fund expenditure, epidemic morbidity rate, and the resident health insurance expenditure as the output indicators, the operational efficiency of the national basic health insurance was measured and evaluated for four years, starting from 2017 when China consolidated its basic health insurance for residents up to 2020, excluding the environmental factors and the random interference, and the main conclusions were drawn as follows.

(1) From the efficiency results, the efficiency values in the third stage differ from those in the first stage, and the number of the efficiency frontier regions decreases from five to four, indicating that the operation of the basic medical insurance is disturbed by the external environment, and it is necessary to exclude the environmental factors when measuring the efficiency values. Following the adjustment, except for two regions (Henan Province and Qinghai Province), which are in the efficiency frontier both before and after the adjustment, the efficiency of the basic medical insurance operations in all other regions has changed, to different degrees, compared with that before the adjustment, and there are great differences in the efficiency of the basic medical insurance fund operations in the different regions of China.
(2) In terms of the environmental factors, the increase of the GDP per capita and the financial investment in the social security and employment, effectively reduce the input redundancy in the operation of the basic medical insurance, which is conducive to improving the efficiency of the medical insurance operation and to better protecting the health of residents. The high level of the GDP represents the economic development, which is conducive to increase the financial investment to improve the development level and speed of health care; the government increases the investment in the social security business, which can fundamentally improve the social security level to drive the high speed development of the medical insurance business; the financial income and expenditure ratio represents the government financial freedom, the higher the financial freedom, the greater the likelihood of the government’s investment in health technicians, which is conducive to the operation of the medical insurance, but it is undesirable to blindly increase this factor, which should make the operation efficiency of the basic medical insurance effectively improved on the basis of moderation.
(3) In a cross-sectional comparison, Guizhou province has the lowest efficiency value of the basic health insurance operation, which, considering the local economic situation, indicates that the economic development affects the investment in health in Guizhou province, which in turn affects the level of social health and the efficiency of the health insurance operation there. However, a blind increase in the investment is not the best decision to improve the efficiency, and some provinces and cities with a good economic development are not in the efficiency frontier after excluding the environmental factors, which indicates that the investment of resources may cause waste, and a reasonable macro coordination is still needed to achieve the best efficiency.
(4) In terms of a longitudinal comparison, the efficiency values of the 31 provinces, from 2017–2020, have been fluctuating erratically, indicating that China’s health insurance operation is still in a developmental stage and has not yet found an applicable and stable solution for the sustainable health insurance development.

## 6. Suggestions

By incorporating the above findings, this study has some management enlightenment and guiding significance for improving the operational efficiency of the basic health insurance funds in China, as well as in the rest of the world. In order to further optimize the allocation of health care resources and improve the operational efficiency of health insurance funds, we propose the following recommendations:

(1) Focusing on the economic construction and increasing the GDP, developing the economy, as the first priority, can improve the expansion of the social capital, enrich the medical talent team, and increase the number of health technicians. By enlarging the financial investment in health, we can improve the social health and health level, and fundamentally improve the efficiency of the health insurance operation. At the same time, we should improve the investment efficiency of the health insurance funds and broaden the investment channels.
(2) While increasing investment in health care, it is more important to improve the level of health care services, reduce the slack variable of the investment, and improve the efficiency driven by quality. First, the government and hospitals should improve the level of management and allocation of resources, taking into account the regional economy, the health service level and other factors, to promote the standardized and high-efficiency development of the health care. Second, the technological innovation should be used to promote a high-quality development, develop technology platforms, develop “Internet+” medical care, improve the ability to monitor the health insurance funds, conduct real-time monitoring of the health insurance funds, and promptly identify problems in the operation of health insurance funds.
(3) Complete the corresponding laws and regulations, improve the level of the health insurance supervision, and facilitate the transparency of the health insurance operation. At the same time, the system of using the medical insurance funds is refined to make the medical insurance system more sound. A robust medical insurance regulation is beneficial for the medical insurance funds to be taken from and used by the people, and to play a role in protecting people’s health, thus improving its operational efficiency. At the same time, the hybrid payment methods can be explored to curb the spike in medical expenditures. Currently there is an inflation of the health insurance fund expenditures, due to fraud, and due to the influential relationship of the payment methods on the irrational use of health insurance funds. China has been reforming its health insurance system in recent years, including the diagnosis-related groups by disease (DRGs) in hospitals, as well as the capitation and prepayment systems in the basic level [2]. Scientific payment methods are the key to control the overuse of services and drugs and the growing health care expenditures, which will drive the reform of the health insurance system [29], so the promotion of hybrid payments for health insurance is necessary.

## 7. Shortcomings and Research Prospects of This Paper

### 7.1. Shortcomings

(1) Since the basic medical insurance for urban and rural residents in China started in 2016, the data were counted from 2017. The operation time is not long enough, and the model used is an efficiency measure for the current situation, so the results obtained, when conducting the empirical model analysis, cannot make a clear judgment on the expected development.
(2) The resident health insurance system contains many elements and has a wide scope. This paper is based on the existing literature, as a reference, and the economic principles, as a guide to measure and analyze, so there are limitations in the selection of perspectives, the relevant indicators and the influencing factors.

### 7.2. Prospect

(1) Continued data collection in subsequent studies may allow for the predictions of efficiency that could better improve the efficiency of the basic health insurance.
(2) Based on this paper, we should continue to conduct a multi-faceted and more in-depth study on the basic medical insurance for urban and rural residents, in order to have a richer and stereoscopic research result. At the same time, since the studies on the operational efficiency of the basic medical insurance for urban and rural residents are still few, there is a need to refer to the relevant research methods in other fields for enrichment.

## Figures and Tables

**Table 1:** Summary of the Input and Output Indicators about the Health Service Efficiency.

Author	Region	Period of Study	Methods	Input Indicators	Output Indicators
Li et al. (2021) [9]	China	2014–2019	Three-stage DEA model	Fund income; Number of insured persons;	Fund expenditure; Number of recipients;
Chiu et al. (2022) [22]	Taiwan	2015–2018	Dynamic data envelopment analysis (DDEA) model	The number of doctors; The number of beds; Equipment expenditure.	Inpatient and outpatient revenue; Earnings; Rate of emergency transfer to the inpatient.
Ngobeni et al. (2020) [23]	South Africa	2017–2018	Data envelopment analysis (DEA)	Total health spending; Total health staff.	IMR (infant mortality rate).
Audibert et al. (2011) [24]	China	2000–2008	Two-stage DEA	The number of staff and equipment.	Number of recipients
Brockett et al. (2004) [25]	The U.S.	1995	Data envelopment analysis (DEA)	Total premium; Total expenses.	Ambulatory encounters; Hospital days.
Chuang et al. (2011) [26]	Taiwan	2006	DEA-ANN approach	The number of patient beds; The number of physicians and nurses; The number of professionals.	The number of inpatient days; The number of outpatient/emergency visits; The in-hospital survival rate.
Patrick et al. (2018) [27]	The U.S.	2014	Data envelopment analysis (DEA)	Health expenditures.	Enrollment; Inpatient admissions; Ambulatory encounters;
Yi et al. (2020) [10]	China	2005–2017	Three-stage DEA model	Financial input (GE); Labor input.	Diagnoses and treatment numbers; Mortality rate; Incidence rates.

**Table 2:** Descriptive Analysis of the Inputs, Outputs and Environmental Variables.

Category	Variables		Mean	Standard Deviation	Minimum	Maximum
Input indicators	Fund income		262.8585484	215.73983	4.70	1561.00
	Number of health technicians		319,278.2903	202,100.02738	16,526.00	829,396.00
	Government health expenditure		524.4196	308.28872	93.80	1772.99
Output indicators	Fund expenditure		229.2443548	170.43871	3.50	682.70
	Epidemic morbidity rate		227.7126613	91.90986	80.80	589.41
	Resident health insurance expenditure		1756.071774	574.78107	271.50	3739.70
Environmental Variables	GDP per capita		66,532.7177	30,122.06163	28,497.00	164,889.00
	Government financial level	Social security and employment expenditure	879.3052	430.11084	107.92	1998.67
		Fiscal revenue expenditure ratio	0.4471	0.18748	0.10	0.88

**Table 3:** Correlation analysis of the inputs and outputs.

	Fund Expenditure	Epidemic Morbidity Rate	Resident Health Insurance Expenditure	Fund Income	Number of Health Technicians	Government Health Expenditure
Fund expenditure	1					
Epidemic morbidity rate	−0.258 ***	1				
Resident health insurance expenditure	−0.100	−0.448 ***	1			
Fund income	0.802 ***	−0.164	−0.107	1		
Number of health technicians	0.899 ***	−0.314 ***	0.035	0.730 ***	1	
Government health expenditure	0.850 ***	−0.216 **	−0.001	0.710 ***	0.925 ***	1

Note: *** and ** indicate the significant *p* value at the 1% and 5%.

**Table 4:** Efficiency of the basic medical insurance funds in China, from 2017–2020.

Province	2017	2018	2019	2020	Mean
Beijing	0.816	0.907	1	0.722	0.861
Tianjin	0.861	1	1	1	0.965
Hebei	0.915	0.936	0.945	0.974	0.943
Liaoning	0.795	0.926	0.877	0.745	0.836
Shanghai	0.981	1	0.795	0.873	0.912
Jiangsu	0.862	0.862	0.933	0.984	0.910
Zhejiang	1	0.999	0.865	0.876	0.935
Fujian	0.87	0.945	0.963	1	0.946
Shandong	1	0.94	0.898	0.944	0.946
Guangdong	0.878	0.94	0.811	0.899	0.882
Hainan	0.875	0.913	0.927	1	0.929
Shanxi	0.851	0.986	1	0.965	0.9505
Jilin	0.875	0.894	1	0.793	0.8905
Heilongjiang	0.866	0.846	0.911	0.764	0.847
Anhui	0.79	0.981	1	1	0.943
Jiangxi	1	1	1	1	1
Henan	1	1	1	1	1
Hubei	0.825	0.914	0.939	0.98	0.915
Hunan	0.966	0.995	0.998	1	0.990
Neimenggu	0.894	1	0.875	0.835	0.901
Guangxi	0.623	0.807	0.953	1	0.846
Chongqing	0.956	0.878	0.887	0.961	0.921
Sichuan	0.823	0.806	0.845	0.935	0.852
Guizhou	0.666	0.976	0.828	0.862	0.833
Yunnan	0.871	0.885	0.891	0.934	0.895
Tibet	1	1	1	1	1
Shanxi	0.97	0.996	0.908	0.742	0.904
Gansu	1	1	0.927	0.91	0.959
Qinghai	1	1	1	1	1
Ningxia	1	1	1	1	1
Xinjiang	0.717	0.984	0.971	0.663	0.834
Mean	0.889	0.946	0.934	0.915	0.921

**Table 5:** Regression results of the SFA model at stage 2.

Variables	Fund Income	Number of Health Technicians	Government Health Expenditure
GDP per capita	−73.78 ***	−38,597.19 ***	−152.38 *
Social security and employment expenditure	−87.84 ***	−58,345.06 ***	−125.65 *
Fiscal revenue expenditure ratio	10.02 ***	−1226.08 ***	6.90 *
Sigma-squared	18,275.43 ***	4,360,611,600.00 ***	6886.31 *
Gamma	0.87 ***	0.53 ***	0.02 *
Log likelihood function	−712.59 ***	−1518.81 ***	−729.46 *
LR test of the one-sided error	124.86 ***	17.89 ***	4.64 *

Note: *** and * indicate significant *p* value at the 1% and 10%.

**Table 6:** Efficiency comparison of the basic medical insurance before and after the adjustment.

	Results of Stage 1			Results of Stage 3		
	TE	PTE	SE	TE	PTE	SE
Beijing	0.86125	1	0.86125	1	1	1
Tianjin	0.96525	1	0.96525	1	1	1
Hebei	0.9425	0.9455	0.9965	0.9235	0.946	0.97625
Shanxi	0.9505	0.95625	0.99325	0.88175	0.9505	0.92825
Neimenggu	0.901	0.9335	0.96475	0.89775	0.9335	0.964
Liaoning	0.83575	0.91175	0.91675	0.906	0.9165	0.98875
Jilin	0.8905	0.9125	0.97475	0.84725	0.92425	0.91825
Heilongjiang	0.84675	0.91075	0.93175	0.89775	0.93475	0.96075
Shanghai	0.91225	0.995	0.91625	0.99275	0.9955	0.99725
Jiangsu	0.91025	0.94125	0.96675	0.93425	0.94075	0.99325
Zhejiang	0.935	0.956	0.97725	0.95275	0.955	0.99775
Anhui	0.94275	0.94725	0.99425	0.9165	0.9575	0.95225
Fujian	0.9445	0.95675	0.987	0.865	0.94075	0.92
Jiangxi	1	1	1	0.97425	1	0.97425
Shandong	0.9455	0.97175	0.973	0.961	0.97175	0.98925
Henan	1	1	1	1	1	1
Hubei	0.9145	0.98675	0.92725	0.975	0.988	0.98675
Hunan	0.98975	0.99625	0.9935	0.99475	0.99625	0.9985
Guangdong	0.882	0.99875	0.88325	0.9695	0.99875	0.9705
Guangxi	0.84575	0.86775	0.96975	0.865	0.86875	0.99625
Hainan	0.92875	0.94525	0.98225	0.8785	0.9155	0.959
Chongqing	0.9205	0.948	0.97125	0.9375	0.9495	0.9865
Sichuan	0.85225	0.9185	0.92825	0.9045	0.919	0.985
Guizhou	0.833	0.84175	0.9885	0.784	0.8355	0.93725
Yunnan	0.89525	0.90775	0.98675	0.858	0.91	0.94325
Tibet	1	1	1	0.91225	1	0.91225
Shanxi	0.904	0.933	0.96625	0.8705	0.937	0.932
Gansu	0.95925	0.962	0.997	0.87875	0.964	0.9105
Qinghai	1	1	1	1	1	1
Ningxia	1	1	1	0.896	1	0.896
Xinjiang	0.83375	0.982	0.8465	0.96425	0.982	0.981

## Data Availability

Data available in a publicly accessible repository. The data presented in this study are openly available in the National Bureau of Statistics at http://www.stats.gov.cn/tjsj/ndsj/, accessed on 12 September 2022.
